# Formation of Multispheres and Myelin Based on Multiple Solutions of Membrane Shape Equation

**DOI:** 10.3390/membranes15100319

**Published:** 2025-10-16

**Authors:** Tao Xu, Zhong-Can Ou-Yang

**Affiliations:** 1 Institute of Theoretical Physics, Academia Sinica, Beijing 100080, China; oy@itp.ac.cn; 2 Institute of Fusion and Plasma Research, College of Electrical and Electronic Engineering, Huazhong University of Science and Technology, Wuhan 430074, China

**Keywords:** membrane, multiple-spheres, myelin

## Abstract

In this work, we construct a multiple solutions theory based on a membrane shape equation. The membrane shape of a vesicle or a red blood cell is determined using the Zhongcan–Helfrich shape equation. These spherical solutions, which have an identical radius $r_{s}$ but different center positions, can be described by the same equation: $\phi -\rho /r_{s}=0$. A degeneracy for the spherical solutions exists, leading to multisphere solutions with the same radius. Therefore, there can be multiple solutions for the sphere equilibrium shape equation, and these need to satisfy a quadratic equation. The quadratic equation has a maximum of two roots. We also find that the multiple solutions should be in a line to undergo rotational symmetry. We use the quadratic equation to compute the sphere radius, together with a membrane surface constraint condition, to obtain the number of small spheres. We ensure matching with the energy constraint condition to determine the stability of the full solutions. The method is then extended into the myelin formation of red blood cells. Our numerical calculations show excellent agreement with the experimental results and enable the comprehensive investigation of cell fission and fusion phenomena. Additionally, we have predicted the existence of the bifurcation phenomenon in membrane growth and proposed a control strategy.

## 1. Introduction

Human red blood cell (RBC) membranes are composed of a fluid lipid bilayer and a triangular network of spectrin tetramers [1,2,3] that is about 4 to 10 nanometers thick. Phospholipids make up 25–30% of the dry weight of the cell protoplasma. There is a high degree of material transport within and between cells via membrane fusion and fission, described as exocytosis and endocytosis. These processes play a central functional role in living systems. The configuration of red blood cells is usually similar to that of a biconcave disk in human beings and a sphere in breastfed animals. But when red cells are profoundly damaged or become necrotic [4], the interaction of phospholipids with the aqueous protein solution gives rise to structures called myelin figures [5]. They are of two types: external and internal structures.

Recently, the phenomenon of budding, i.e., the expulsion of a smaller vesicle out of a larger one, has attracted a lot of interest [6]. Many response experiments of single-component giant unilamellar vesicles (GUVs) subjected to different external osmotic stresses have been made. The mutual adhesion of giant vesicles can be studied using micropipette aspiration techniques [7,8,9]. It has been found that giant vesicles can transform into different multispheres; the small spheres can be external or internal. This process is similar to the myelin formation of red blood cells. In fact, these shape transformations can be unified, described, and computed using the membrane theory of Ou-Yang Zhong-Can and Helfrich [10].

This study is organized as follows: The membrane shape equation is given in Section 2. The multisphere solution is presented in Section 3. The simulation results based on experiments performed using our method are presented in Section 4. The myelin formation is discussed in Section 5. Finally, the main conclusions are summarized in Section 6.

## 2. Membrane Shape Equation

In the year 1973, W. Helfrich proposed that the shapes of vesicles or red blood cells satisfy free bending energy [11]:

(1) $$F=\frac{1}{2}k_{c}\oint {\left(c_{1}+c_{2}-c_{0}\right)}^{2}ds+\bar{k}\oint c_{1}c_{2}ds+\Delta p\int dv+\lambda \oint ds,$$

where $k_{c}$, $\bar{k},$$c_{1}$, $c_{2}$, and $c_{0}$ are the bending rigidity, Gaussian curvature modulus, two principal curvatures, and spontaneous curvature, respectively. $\Delta p=p_{0}-p_{i}$ is the osmotic pressure difference between the outer and inner media, and λ is tensile stress.

Ou-Yang Zhong-can et al. minimized the variation in free energy, i.e., δF=0, and obtained the equilibrium shape equation as [10,12,13,14]

(2) $$k_{c}\nabla^{2}\left(2H\right)+k_{c}\left(2H+c_{0}\right)\left(2H^{2}-2K-c_{0}H\right)-2\lambda H+\Delta p+{\bar{\nabla }}^{2}\bar{k}=0,$$

where *H* and *K* are the mean curvature and Gaussian curvature defined as

(3) $$H=-\frac{1}{2}\left(c_{1}+c_{2}\right),\;K=c_{1}c_{2}.$$

here, $c_{1}$ and $c_{2}$ are principle curvatures of the membrane surface.

It is obvious that Equation (2) has a sphere solution with radius $r_{0}$, which satisfies

(4) $$\Delta pr_{0}^{3}+2\lambda r_{0}^{2}-k_{c}c_{0}r_{0}\left(2-c_{0}r_{0}\right)=0.$$

If a vesicle has a constant Gaussian curvature modulus, then ${\bar{\nabla }}^{2}\bar{k}=0$. The shape equation becomes [10]

(5) $$k_{c}\nabla^{2}\left(2H\right)+k_{c}\left(2H+c_{0}\right)\left(2H^{2}-2K-c_{0}H\right)-2\lambda H+\Delta p=0.$$

**Figure 1:**
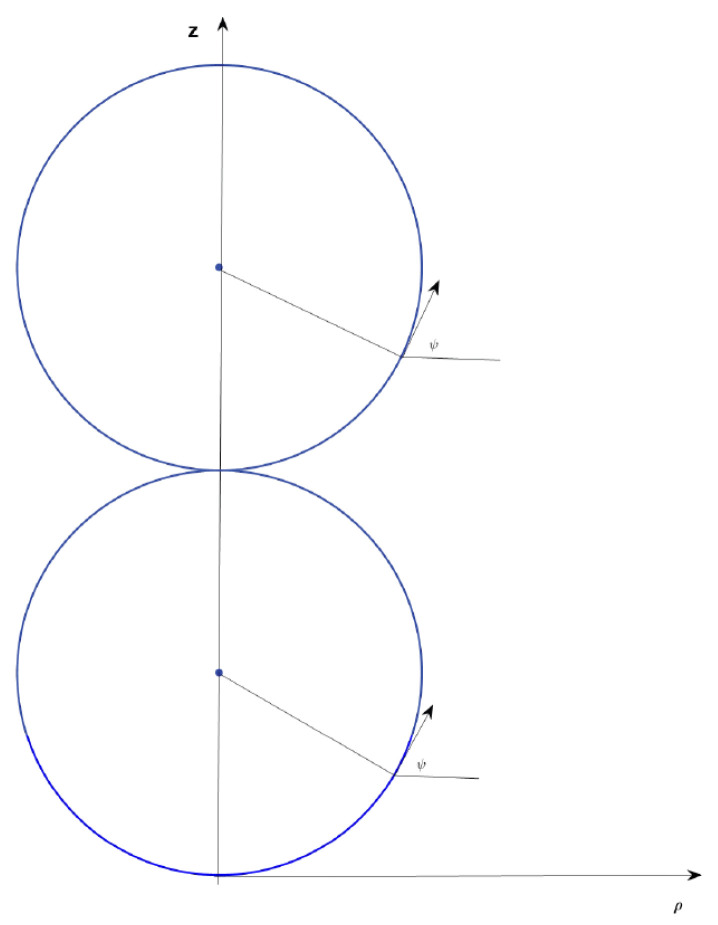
Coordinates for the description of axisymmetric shapes. The symmetric axis of membrane surface is *Z*-axis. The radial distance from the surface to the *Z*-axis is ρ. The angle between the tangent line of the contour at the point and the ρ axis is ψ. There are small spherical vesicles that are lined up one next to the other in a straight row. The straight line is oriented along the *Z*-axis. All of these spherical solutions, which have an identical radius r but different center positions shown can be described by the same equation sin(ψ) = ρ/r.

According to tradition, we choose (X,Y,Z) as the coordinate of a point on the membrane surface. If the surface is axisymmetric, we select the symmetric axis as the *Z*-axis. The radial distance from the surface point to the *Z*-axis is ρ. The angle between the projection of the point onto the base X−Y plane and positive *X*-axis is defined as the azimuthal angle θ. The angle between the tangent line of the contour at that point and the ρ-axis is ψ. Then, X=ρcos(θ), Y=ρsin(θ), and dZ=tan(ψ)dρ. The coordinates system used is shown in Figure 1.

The axisymmetric membrane shape Equation (5) can be simplified as [15]

(6) $$\begin{array}{ccc} & & -\frac{\cos\psi }{\rho }\{\rho \cos\psi [\frac{{\left(\sin\psi \right)}^{\prime }}{\rho }{]}^{\prime }{\}}^{\prime }-\frac{1}{2}[\frac{{\left(\rho \sin\psi \right)}^{\prime }}{\rho }{]}^{3}+\\ & & \frac{{\left(\rho \sin\psi \right)}^{\prime }{\left(\sin^{2}\psi \right)}^{\prime }}{\rho^{2}}-\frac{c_{0}{\left(\sin^{2}\psi \right)}^{\prime }}{\rho }+\frac{\bar{\lambda }{\left(\rho \sin\psi \right)}^{\prime }}{\rho }+\bar{p}\\ & = & 0, \end{array}$$

where $\bar{\lambda }=\lambda /k_{c}+c_{0}^{2}/2$, $\bar{p}=p/k_{c}$. In the present study, we use the ${\mathrm{symbol}\;}^{\prime }$ to represent differentiation $\frac{d}{d\rho }$.

Let ϕ=sinψ. By multiplying both sides of the above equation by ρ and applying the following equation

(7) $$-\frac{\rho {\left(\phi^{2}\right)}^{\prime }}{2}[\frac{{\left(\rho \phi \right)}^{\prime }}{\rho }{]}^{\prime }-\frac{\rho }{2}[\frac{{\left(\rho \phi \right)}^{\prime }}{\rho }{]}^{3}+\frac{{\left(\rho \phi \right)}^{\prime }{\left(\phi^{2}\right)}^{\prime }}{\rho }=[\frac{\phi^{3}-\phi {\left(\rho \phi^{\prime }\right)}^{2}}{2\rho }{]}^{\prime },$$

we can obtain

(8) $$\{\frac{\phi^{3}-\phi {\left(\rho \phi^{\prime }\right)}^{2}}{2\rho }-\rho \left(1-\phi^{2}\right)[\frac{{\left(\rho \phi \right)}^{\prime }}{\rho }{]}^{\prime }-c_{0}\phi^{2}+\bar{\lambda }\rho \phi +\frac{\bar{p}\rho^{2}}{2}{\}}^{\prime }=0.$$

The above equation can be written in the first integral form [16]

(9) $$\rho \left(\phi^{2}-1\right)\phi^{\prime \prime }-\frac{\phi \rho }{2}{\left(\phi^{\prime }\right)}^{2}+\left(\phi^{2}-1\right)\phi^{\prime }+\frac{\phi }{\rho }-\frac{\phi^{3}}{2\rho }-c_{0}\phi^{2}+\bar{\lambda }\rho \phi +\frac{\bar{p}}{2}\rho^{2}+\eta_{0}=0,$$

where $\eta_{0}$ is an integral constant.

Let us assume a characteristic length $r_{s}$ for membrane shape. Let $\rho =r_{s}\rho_{s}$, ${\bar{\lambda }}_{s}=\bar{\lambda }r_{s}^{2}$, ${\bar{p}}_{s}=\bar{p}r_{s}^{3}$, $c_{0s}=c_{0}r_{s}$, $\eta_{0s}=\eta_{0}r_{s}$, then we obtain

(10) $$\rho_{s}\left(\phi^{2}-1\right)\frac{d^{2}}{d\rho_{s}^{2}}\phi -\frac{\phi \rho_{s}}{2}(\frac{d}{d\rho_{s}}\phi {)}^{2}+\left(\phi^{2}-1\right)\frac{d}{d\rho_{s}}\phi +\frac{\phi }{\rho_{s}}-\frac{\phi^{3}}{2\rho_{s}}-c_{0s}\phi^{2}+{\bar{\lambda }}_{s}\rho_{s}\phi +\frac{\bar{p_{s}}}{2}\rho_{s}^{2}+\eta_{0s}=0.$$

The above equation has a sphere solution $\phi =\rho /r_{s}$ with condition $\eta_{0s}=0$. The sphere radius $r_{s}$ is obtained using the above equation:

(11) $$\frac{{\bar{p}}_{s}}{2}r_{s}^{2}+\bar{\lambda_{s}}r_{s}-c_{0s}=0.$$

If p=0, $\bar{\lambda_{s}}\neq 0$, the above equation has solution $r_{s}=c_{0s}/\bar{\lambda_{s}}$. If p≠0, the above equation has the following solutions:

(12) $$r_{s\pm }=-\frac{\bar{\lambda_{s}}}{{\bar{p}}_{s}}\pm \frac{\sqrt{\bar{\lambda_{s}}+2{\bar{p}}_{s}c_{0s}}}{{\bar{p}}_{s}}.$$

Equation (11) has a single solution $r_{s}=r_{s1}$ when p=0, $\bar{\lambda_{s}}=0$ or $\bar{\lambda_{s}}+2{\bar{p}}_{s}c_{0s}=0$.

Many cellular transport processes involving the plasma membrane include different forms of endocytosis [17,18], exocytosis [19,20], and vesicle budding from intracellular organelles [21,22]. These biological processes can be explained by the solution of Equation (11). If the two roots of this quadratic equation are imaginary—that is, there is no spherical solution—it might correspond to the biconcave red blood cell solution; if one is positive and one is negative, this would likely indicate endocytosis [23], and if both roots are positive, this would likely indicate exocytosis [24].

## 3. Multisphere Solution

In this section, we provide a realistic computation for the multisphere configuration for vesicles.

If $r_{s+},r_{s-}$ are non-zero solutions of Equation (11), quadratic Equation (11) can be factored into an equivalent equation of the following form:

(13) $$\left(r_{s}-r_{s1}\right)\left(r_{s}-r_{s2}\right)=0.$$

here, $r_{s1}=r_{s+}$, $r_{s2}=r_{s-}$. If Equation (11) has a single solution, then the above equation becomes $r_{s}-r_{s1}=0$.

If the membrane shape equation admits a spherical solution of radius $r_{s1}$, then the solution can be written as $\phi -\rho /r_{s1}=0$. It can be written in (X,Y,Z) coordinates, i.e.,

(14) $$X^{2}+Y^{2}+{\left(Z-z_{i}\right)}^{2}=r_{s1}^{2}$$

where ($0,0,z_{i}$) are the coordinates of the sphere center. For different values of $z_{i}$, this equation represents spheres with different center positions on the *Z*-axis, although all of these spheres have the same radius $r_{s1}$.

There are small spherical vesicles that are lined up one next to the other in a straight row. The straight line is oriented along the *Z*-axis. All of these spherical solutions, which have an identical radius $r_{s1}$ but different center positions shown in Figure 1, can be described by the same equation

(15) $$\phi -\rho /r_{s1}=0.$$

By virtue of the (ρ,ψ) coordinates, the multisphere solution can be written as

(16) $${\left(\phi -\rho /r_{s1}\right)}^{N_{1}}=0,$$

where these spheres have the same radius $r_{s1}$ with a different center position on the *Z*-axis. The non-negative integer number $N_{1}$ presents the number of spheres with the same radius.

Due to the symmetry of the axisymmetric membrane shape equation, a degeneracy exists for the spherical solutions, leading to multisphere solutions with the same radius. Multiple solutions need to satisfy quadratic Equation (11). The quadratic equation has a maximum of two roots. Hence, general multisphere solutions can be written as

(17) $${\left(\phi -\rho /r_{s1}\right)}^{N_{1}}{\left(\phi -\rho /r_{s2}\right)}^{N_{2}}=0,$$

where degeneracy numbers $N_{1}$, $N_{2}$ are non-negative integers. These spheres of different radii are arranged one after another along the *Z*-axis to form a line. All multisphere solutions should satisfy the same constraint (13). This means that the equation has $N_{1}$ spheres with radius $r_{s+}$, and $N_{2}$ spheres with radius $r_{s-}$.

If the cell membrane equation has a unique solution, the membrane remains unchanged due to the conservation of the membrane area. However, if the equation admits two distinct solutions, the cell may develop functional abnormalities. Hence, we focus on multispheres with two different radii in this study.

Radii $r_{s1}$ and $r_{s2}$ satisfy the relation

(18) $$\frac{1}{r_{s1}}+\frac{1}{r_{s2}}=\frac{\tilde{\lambda }}{c_{0}}.$$

These spheres should be in a line under the condition of rotational symmetry. It is clear that the sphere solution of $\phi -\rho /r_{s1}=0$ or $\phi -\rho /r_{s2}=0$ is on the *Z*-axis. The symmetric research of differential equations has been analyzed by Lie’s group [25]. Lie’s group’s analysis of the axisymmetric membrane shape equation is discussed in [26].

Equation (10) has a general sphere solution when $p=\lambda =c_{0}=\eta =0$. The radius of the sphere can be arbitrarily large in this special condition, but the minimization of bending energy drives small spheres into a single larger sphere.

Giant vesicles can form shapes that consist of multispheres connected by narrow membrane necks. Within the Helfrich spontaneous curvature model, such shapes arise quite naturally and can be reached by the deflation of smoothly curved shapes. In this study, we consider a giant vesicle with radius $R_{s}$. It transforms into $N_{1}$ same-sphere vesicles with radius $r_{1}$ and $N_{2}$ small same-sphere vesicles with radius $r_{2}$. We define $r_{1}\geq r_{2}$. The original volume of giant vesicles is $V_{s}=\frac{4}{3}R_{s}^{3}$, and the volume of all small spheres is *V*. The relative volume parameter is $\nu =\frac{V}{V_{s}}\leq 1$. If the total area of the membrane is almost unchanged, then

(19) $$1=N_{1}r_{s+}^{2}+N_{2}r_{s-}^{2},\;\nu =N_{1}r_{1s}^{3}+N_{2}r_{2s}^{3}.$$

This result was first obtained by R. Lipowsky et al. [27]. For more theoretical analysis and experimental results, one can refer to R. Lipowsky’s book, *Understanding giant vesicles*, which is Reference [28].

The energy variation satisfies

(20) $$F_{0}\geq N_{1}F_{1}+N_{2}F_{2},$$

where $F_{0}$ is the energy of the original vesicle before undergoing shape transformation, and $F_{1}$ and $F_{2}$ are the energies of the other two types of vesicles after undergoing shape transformation.

The Gaussian curvature modulus $\bar{k}$ is about $1.0\times 10^{-19}$ J, which resists topological change. The formation of a membrane neck is mediated by multiple biochemical mechanisms, including mechanoenzymes belonging to the dynamin family [29], helix insertion due to BAR domain proteins [30], and ESCRT proteins [31,32]. A common organizational feature of these different proteins is that they form helical assemblies at the membrane neck through oligomerization [33,34]. The range of forces and deflection over which they operate suggests that the formation of the membrane neck is functionally mechanical [28,35,36,37] and robust to changes in the biological environment.

The budding transition is the most thoroughly studied shape transformation. Its appeal originates not only from the close resemblance to biologically important phenomena but also from an extensive number of physical experimental data [38]. There are several theoretical models of budding transition; for example, the bilayer couple model [39] and the spinodal fluctuations model [40]. In fact, budding transition is a time-dependent dynamic process. In this study, we provide a phenomenological approximation model for the membrane neck.

Multispheres are closely connected, but thermal fluctuations in the surrounding liquid induce stretching. Then, small spheres are distorted and necks are formed. Above a critical temperature, excess thermal energy destabilizes the vesicles’ topology via increased fluctuation amplitudes. Below the critical temperature, the small vesicles are linked by $N_{1}+N_{2}-1$ necks with the following lengths [12]:

(21) $$L_{i}=z_{i,1}+z_{i,2},\;i\leq N_{1}+N_{2}-1.$$

The maximum opening radius of the neck, the perpendicular distance from the neck’s circular boundary to the *z*-axis, is defined as *D*. The shape of the necks can be written approximately as [41]

(22) $$D_{1}=a\cosh(\left(z_{1}-z_{0}\right)/a,\;D_{2}=a\cosh\left(\left(z_{2}-z_{0}\right)/a\right),$$

where $D_{1}\leq r_{1}$, $D_{2}\leq r_{2}$, $\;z_{2}\leq z_{0}\leq z_{1}$. Here, *a* is the minimum non-zero radius that is determined by a balance between the membrane lateral tension and bending rigidity: $a=\sqrt{k_{c}/2\sigma }$ [10,42,43,44]. Here σ is the tension around the sphere. This formular is derived in detail in reference [44]. It can be also obtained from reference [10,13]. Two spheres is connected by a neck. The critical pressure $P_{c}$ satisifies

(23) $$P_{c}=\frac{2k_{c}}{r^{3}}\left[l\left(l+1\right)-c_{0}r\right],$$

where *l* is the degree order number of spherical harmonics $Y_{lm}\left(\theta ,\varphi \right)$ for deformation perturbation, and *r* is the radius of a big sphere. If the neck is a circular cylinder with radius *a*, then

(24) $$P_{c}a^{3}+\left(\lambda +\frac{1}{2}k_{c}c_{0}^{2}\right)a^{2}-\frac{1}{2}k_{c}=0.$$

If r≫a, then $P_{c}a^{3}/k_{c}\ll 1/2$. Then one can obtain

(25) $$a\approx \sqrt{k_{c}/2\lambda^{\prime }},$$

where $\lambda^{\prime }=\lambda +\frac{1}{2}c_{0}^{2}k_{c}\approx \frac{1}{2}c_{0}^{2}k_{c}\approx \sigma$ [44]. This result is the same as W. Helfrich’s result in reference [44]. This formula is a good approximation, and Formular (24) is recommended for an accurate calculation of *a*. Furthermore, it should be noted that the neck is not cylindrical but is employed to approximate the radius at its endpoint in this paper.

If $k_{c}=1.0\times 10^{-19}$ J, σ=0.001 ${\mathrm{Nm}}^{-1}$, *a* is about 7.1 nm. If a=0, then the sphere will break and the topological number $\oint c_{1}c_{2}ds$ of the vesicles will be changed. In addition, if the thickness of the lipid membrane is considered, the sphere will break when *a* calculated is less than the thickness of the membrane.

## 4. Multisphere Formation in GUV Experiments

To investigate the multisphere transition mechanism, many GUV experiments have been performed in different laboratories. It is known that slight external perturbations can break the equilibrium and result in membrane morphological remodeling. For example, temperature variations have resulted in pear, discocyte, or stomatocyte shapes transitioning to budding [45]. It has also been found that the key shape transition is membrane asymmetry, which can be driven by molecular densities or components [46,47,48,49].

The experimental studies in Ref. [27] consider lipid vesicles exposed to two types of simple sugars: glucose and sucrose. Their experimental procedure involved several steps. Firstly, the vesicles were prepared in aqueous solutions that contained only 234 mM of sucrose. After this preparation step, both leaflets of the vesicle membrane were exposed to the same sucrose concentration. Subsequently, a small amount of the vesicle–sucrose solution was transferred into the observation chamber with 219 mM of glucose plus 15 mM of sucrose. Thus, after this transfer step, the resulting asymmetry between the two bilayer leaflets created a striking variety of multispherical or “multi-balloon” vesicles, consisting of a variable number of large and small spheres connected by very narrow, hourglass-shaped membrane necks. These necks have a diameter of about 14 nm, which is larger than twice the bilayer thickness. Many labs have generated the same results: Firstly, with each such shape, the radii of the individual spheres could attain at most two different values. Secondly, the multispherical shapes were either composed of large and small spheres with two different radii or, alternatively, several equally sized spheres [27]. This evidence proves the correctness of our theory in this study.

Our approximation calculation methodology for the multispheres with $N_{1}$ large and $N_{2}$ small sphere is detailed below.

If the parameters such as ΔP or $c_{0}$ can be measured in experiments, we can obtain other parameters according to Equation (11). But we do not have enough information about these parameters, then we need to estimate them.

First, spontaneous curvature $c_{0}$ is determined from the minimum radius $r_{\min}$ of two spheres, i.e., $c_{0}$ is approximated to $1/r_{\min}$ if the small spheres is looked as a cylinder. Here radius $r_{\min}$ is measured from experimental data or figures. Then based on Equation (11), we obtain

(26) $$\frac{\Delta P}{c_{0}}=-\frac{2k_{c}}{r_{1}\times r_{2}},\;\lambda =-\frac{1}{2}\left(r_{1}+r_{2}\right)\Delta P-\frac{1}{2}k_{c}c_{0}^{2}\Delta P.$$

One can choose $c_{0}\to 1/a$ where *a* is the radius of a neck if the effect of a neck is emphasized. In this case, the λ is approximated to zero, then one can obtain $c_{0}\approx \frac{2(r_{1}+r_{2})}{r_{1}*r_{2}}$.

Now, let us provide a phenomenological computation for Figure 8C in Ref. [27]. The above shape transformations of vesicles are dynamic processes. Here, we focus on the original and final equilibrium configuration of vesicles. A giant vesicle POPC and 10% mol cholesterol are prepared in 234 mM of sucrose. The giant vesicle has an original radius of $R_{s}\approx$ 5 μm. Then, it is transformed to the observation chamber with 219 mM of glucose plus 15 mM of sucrose. All experiments are performed at room temperature (23 °C).

In order to calculate the radius and number of small spheres, we let $k_{c}\approx 1.5\times 10^{-19}$ J, ΔP=−60 mPa, $\lambda =1.0\times 10^{-7}{\mathrm{Nm}}^{-1}$, $c_{0}=0.90\times 10^{6}\;m^{-1}$. The radius of small spheres is obtained from Equation (12). We obtain $r_{1}=4.5$ μm and $r_{2}=1$ μm. It is obvious that the number of small spheres satisfies Equation (19). Then, we obtain $N_{1}=1$, $N_{2}=4$.

**Figure 2:**
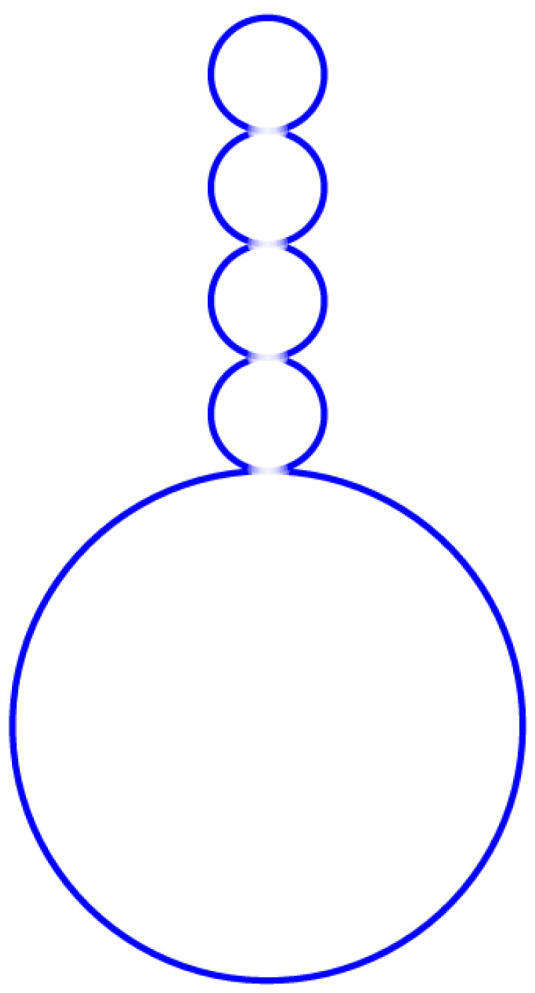
A giant vesicle was prepared in 234 mM sucrose and then transferred into an observation chamber filled with 219 mM glucose plus 15 mM sucrose. A large sphere and four same sized small spheres form a connected symmetric configuration. The radius of the large sphere is 4  μmm, and the radius of each small sphere is 1.0 μmm.

We make use of Equation (22) and let $z_{1}=0.25r_{2}$, $z_{2}=0.25r_{2}$, $\lambda^{\prime }=\lambda +\frac{1}{2}c_{0}^{2}k_{c}$, where $\frac{1}{2}c_{0}^{2}k_{c}$ is the intrinsic tension of curvature elasticity. Then, the minimum radius of the neck between two small spheres is about 0.67 μm. The maximum opening radius of the neck is $D_{1}=D_{2}\approx 0.72$ μm. Due to insufficient data in Figure 8C in Ref. [27], we do not compute the neck region connecting the large and small spheres. But these neck areas do not differ much in terms of their geometric configuration. The above multisphere configuration simulated by our theory is shown in Figure 2. By substituting these parameters into the energy criterion Equation (20), we find that these solutions are stable. It is evident that the bending energy in the neck region between the large and small spheres significantly exceeds that between the small spheres. Energy minimization drives small vesicles to form adjacent to large vesicles within the established neck region. But if a defect occurs locally in a large vesicle, it may induce the relocation of small sphere growth sites and even trigger branching phenomena. We hope to experimentally validate this in future studies.

We have also calculated other figures in [27,49,50]. Our computation results correlate well with the results of these experiments. The solution discriminant $\bar{\lambda_{s}}+2{\bar{p}}_{s}c_{0s}\geq 0$ and energy criterion (20) provide a wide range of parameters. This implies that GUVs with the same membrane area and the same initial volume can attain many different multispherical shapes. The molecular mechanism of the spontaneous curvature could be either a different chemical environment on both sides of the membrane or a different chemical composition of the two monolayers [51,52].

## 5. Myelin Formation

Now, let us consider the myelin formation of red blood cells. If we do not consider the flow of blood in vessels, normal red blood cells have a biconcave disk with ΔP=0. The spectrin network can be isolated by dissolving the lipid with detergents. It then becomes nearly spherical with $c_{0}\geq 0$. When red blood cells begin necrosis, the interaction of phospholipids with the aqueous protein solution gives rise to structures called myelin figures with ΔP≠0.

**Figure 3:**
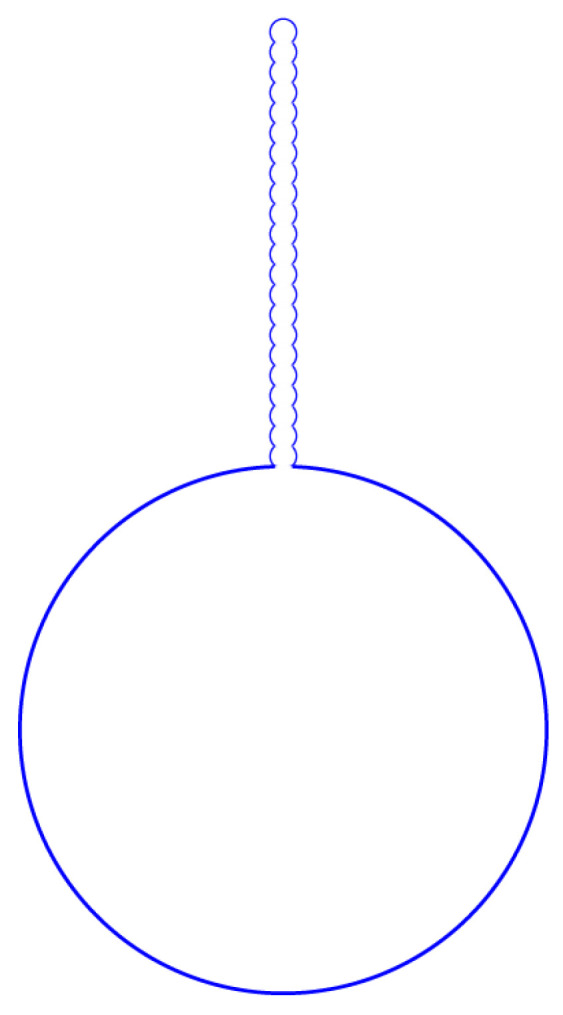
A myelin configuration with topological connectivity is formed by one sphere of radius 3.0 μm and 22 spheres of radius 0.15  μm. 22 small spheres are aligned in a straight line and connected to one large sphere. This myelin formation is simulated by our model as a result of in vitro aging of blood.

The original, normal red blood cells have a size of about $R_{s}=3.1$ μm. When red blood cell necrosis occurs, a new equilibrium is established. For the bending curvature module, $k_{c}\approx 1.6\times 10^{-19}$ J, $\lambda =3.3\times 10^{-6}$ Nm. If ΔP=−3 Pa, $c_{0}=4.2\times 10^{6}m^{-1}$; then, sphere-like red blood cells can transfer into a large sphere with $r_{1}=3$ μm and 22 small spheres with $r_{2}=0.15$ μm, $z_{1}=0.25r_{2}$, and $z_{2}=0.25r_{2}$. The minimum radius of the neck between two small spheres is about 0.13 μm, and the maximum opening radius of the neck is $D_{1}=D_{2}\approx 0.14$ μm. This is the result of Figure 120 in Ref. [5], which shows the myelin forms arising from echinocytes. Our myelin configuration is shown in Figure 3.

Now, let us consider the flow of blood in the vessels. A uniform velocity *U* of red blood cells relative to blood in the capillaries is assumed, and the blood’s viscosity is μ. The blood can be approximated as Newtonian fluid. The stresses on the fluid element per unit volume of incompressible blood are denoted as

(27) $$\Pi =-P_{b}I+\mu \left[\nabla V+{\left(\nabla V\right)}^{T}\right],$$

where V is the velocity of blood and $P_{b}$ is the blood pressure caused by the relative motion of cells. The sphere-like red blood cells have obtained external stress $\sigma_{bn}$ along the radial direction n

(28) $$\sigma_{bn}=\Pi \cdot n.$$

Sphere-like red blood cells are acted upon by external blood pressure [24,53]

(29) $$P_{b}={\left(\sigma_{bn}\cdot n\right)}_{out}-{\left(\sigma_{bn}\cdot n\right)}_{in}=1.5\mu U\cos\left(\theta \right)/R.$$

here, *R* is the radius of the cell, and θ is the angle from the *Z*-axis. The external blood pressure $P_{b}$ is about 10${-}^{6}\cos\left(\theta \right)/R$ in capillaries. The shape equation becomes

(30) $$\bar{P}-\frac{2c_{0}}{R^{2}}+\frac{2\bar{\lambda }}{R}=\bar{P_{b}},$$

where $\bar{P_{b}}=P_{b}/k_{c}$. Here, $\bar{P_{b}}=\frac{1.5\mu U}{k_{c}R}\cos\left(\theta \right)$. Consequently, spherical cells adapt by either deforming to resist the excess pressure or by regulating their surface tension. The term $\bar{\lambda_{s}}r_{s}$ in Equation (11) will add a term −1.5μUcos(θ). The shape equation becomes

(31) $$\bar{P}-\frac{2c_{0}}{R^{2}}+\frac{2\bar{\lambda_{2}}}{R}=0,$$

where $\bar{\lambda_{2}}=\lambda /k_{c}+c_{0}^{2}/2-1.5\mu U\cos\left(\theta \right)$. This situation is analogous to perturbations described by spherical harmonics $Y_{l,m}$, where l=1, m=0 [10]. In the steady state, if the velocity *U* of red blood cells relative to blood becomes zero, the external blood pressure $P_{b}$ turns to zero.

## 6. Conclusions

In conclusion, due to the symmetry of the axisymmetric membrane shape equation, a degeneracy for the spherical solutions exists, leading to multisphere solutions with the same radius. Multiple solutions ${\left(\phi -\rho /r_{i}\right)}^{N_{i}}{\left(\phi -\rho /r_{j}\right)}^{N_{j}}=0$ need to satisfy the same constraint condition (11) or nontrivial constraint condition (4) based on the symmetry of the membrane shape equation, which is a quadratic equation that has a maximum of two roots. The difference in the roots means that a giant vesicle transforms into many different small spheres with only two radius sizes. A double root makes a giant vesicle transform into many small spheres of equal size. The number of small spheres is determined by surface constraint (19). These small spheres should be in a line in order to undergo rotational symmetry. Our method is different from the method of H.J. Deuling et al. [54]. We have applied our theory to the processes of vesicle fusion and fission. We find that our calculation results closely match those of the experimental observations. We have also applied our theory to explain the mechanism of myelin formation in red blood cells. Based on our theory, we can predict the osmotic pressure difference between outer and inner membranes as well as spontaneous curvature. Moreover, we have predicted that a bifurcation phenomenon occurs with the growth of a small sphere-like membrane shape; furthermore, we have proposed a control scheme. Energy minimization drives small vesicles to form adjacent to large vesicles within the established neck region. But if a defect occurs locally in a large or small vesicle, it may induce the relocation of small sphere growth sites and even trigger branching phenomena. If the cell membrane equation admits two distinct solutions, the cell shape has multiple solutions, and the cell may develop functional abnormalities. Our work provides key insights into cell exocytosis and endocytosis, and provides critical evidence of cell mechanism biological development.

## Data Availability

The original contributions presented in this study are included in the article. Further inquiries can be directed to the corresponding author.
